# Prognostic factors of first birth interval using the parametric survival models

**Published:** 2014-02

**Authors:** Zahra Shayan, Seyyed Mohammad Taghi Ayatollahi, Najaf Zare, Fariba Moradi

**Affiliations:** 1 *Department of Biostatistics, Medical School, Shiraz University of Medical Sciences, Shiraz, Iran.*; 2 *Department of Biostatistics, Infertility Research Center, Shiraz University of Medical Sciences, Shiraz, Iran.*; 3 *Department of Family Health and Population, Shiraz University of Medical Sciences, Shiraz, Iran. *

**Keywords:** *First birth interval*, *Survival analysis*, *Parametric models*

## Abstract

**Background:** In recent years, the study of birth intervals has been a main determinant of the levels of fertility in the populations, as it is associated with rates of fertility and population growth.

**Objective: **The purpose of this study was to firstly apply survival analysis for modeling of first birth interval and secondly to explore its determinants.

**Materials and Methods:** In a cross sectional study, the fertility history of 858 women was collected in rural areas of Shiraz (southern Iran) in 2008. We used the survival analysis such as cox regression and alternative parametric models to evaluate the prognostic factors of first birth interval.

**Results:** Among the explanatory variables of interest, age at marriage, level of women's education, and menstrual status had highly significant effects on the duration of birth interval after marriage (p<0.01).

**Conclusion:** It is concluded that the suitable parametric models would be a useful tool for fitting to first birth interval, the fact that has been less paid attention to in researches.

## Introduction

The study of the dynamics of timing and spacing of births is important for several reasons, including an understanding of completed family size as well as maternal and child mortality (1). Modeling fertility data is one of the greatest interests in population economic studies. Several indicators are used to measure fertility patterns, such as the first birth interval after marriage (2). Many theoretical approaches have been developed to explain variations in fertility. The most common measure of fertility is the total fertility rate that is defined as the average number of births that a woman would have if she survived to the end of her childbearing years (3). 

Singh *et al* demonstrated in their study of human reproduction, measurement of fecund ability has got an important place. Among the various types of fertility data used for the purpose, data on first birth interval have an upper hand over all other types of birth interval due to certain reasons. First, being the earliest and first event of the married life of a female, it hardly suffers from recall lapse; second, it is free from the period of post-partum amenorrhea (ppa) associated with a live birth. Other birth intervals are heavily affected by the erratic fluctuations of this period (4). 

Therefore, it is important to study the variations in fertility such as timing between marriage and the first live birth. Different studies have identified different risk factors contributing to the length of birth intervals. Mother’s education and age at marriage are the most widely determinants of birth intervals. Age at marriage of mothers is considered to be an important variable in the fertility process which is negatively associated with the length of birth interval (5-7). Age at marriage may have a varied effect on different birth intervals. West found that the first birth is an important determinant at the transition from parity one to parity two. For older women, he found its importance at the transition from parity two to parity three. Interestingly, his findings also showed that the younger a woman is at first birth, the higher the transition probability (8). Also, education has always been an important variable in the sociological and economic literature of fertility (9). Rasekh and Momtaz stated the encouraging women for higher education and giving opportunity to them to get employed may be the influential way of slowing down fertility in Ahvaz, Iran (10).

Birth spacing has become a main strategy of the health promotion program for mothers and children over the past two decades in Islamic Republic of Iran. In Iran, the effect of the family planning program on the first birth is not significant and it is not effective in delaying the first birth, but it plays a major role in delaying or stopping other births (11). Most of the researches have been carried out in Iran based on modeling of the birth spacing than first birth (12-15). 

So far, a study has not been published on modeling of the timing between marriage and first live birth. It is, therefore, important to study the interval between age at marriage and first birth according to introduced causes. This research has focused on the onset of the family building process, that is, the transition to first birth by using survival models. The purpose of the current study was to, firstly, find the best parametric survival models to analyze first birth interval after marriage and secondly to investigate which factors affect first birth interval.

## Materials and methods

**Study population**

In a cross sectional study, the fertility history of 858 women aged 15-49 years in rural areas of Shiraz (southern Iran) was reviewed (16). The people in such areas are not highly educated, mostly of primary education. The women were selected by multistage random sampling from a list of village Health Houses in 2008. In first stage, 18 Health Houses were selected, then, in second stage, the women were selected randomly within each Health House. Data were collected using a series of forms completed during face-to-face interviews. The form included demographic characteristics and fertility history. The women who were not agreed to participate in this research were excluded. Only the history of the first pregnancy of each woman was included in this study. Therefore, the final sample was including 654 women. Unwanted pregnancy can be one of the main motivations of abortion; therefore, it can confound the result of pregnancy (17-18). For this reason, women with an unwanted first pregnancy were excluded from the study; therefore, the final study sample consisted of 654 women. Furthermore, within a rural subsistence based society, the economic conditions of any household did not differ much. Generally speaking, the subjects were not working hard in the fields. They were also normally free from smoking habits, alcohol, or drug use due to religious commitments. The response variable for analysis is defined the first birth interval. 

Live birth in the first delivery was the success event and a stillborn fetus or abortion (5% in this data set) was considered as censored. The event time was defined as the interval between marriage and a live birth or censoring (years). The explanatory variables were age at marriage, age at first pregnancy, age at menarche, education level of women, education level of husbands, family income, and menstrual cycle. Data were obtained from a university approved project and all ethical consideration has been met. 

**Statistical analysis**

Because the study was included the censored data, the survival analysis methods was carried out. Log rank test was used to compare the survival between different groups. The Cox proportional hazards model, a popular mathematical model, was used to determine the effects of various factors on the first birth interval. This model is usually written in terms of the hazard model formula. In cox model, we can exponent ate the coefficients to obtain hazard ratio to calculate the factor change in the hazard rate associated with the changes of variable. 

The hazard ratio greater than one increases the hazard rate of duration of live birth. The hazard ratio less than one decrease the hazard rate of duration of live birth. The statistical significance of the coefficient indicates whether these changes in expected duration will be statistically significant or not. Moreover, we evaluated the two parametric survival models (Log-logistic and Generalized Gamma), because the cox model is a semi-parametric model. A major advantage of the parametric survival model on the Cox model is that a description of the hazard rate of live birth over time can be plotted. Also, the parametric models tend to give more precise estimates of coefficients. In parametric models, we measure the direct effect of the explanatory variables on the survival time instead of hazard. 

This characteristic allows for an easier interpretation of the results because the parameters measure the effect of the correspondent covariate on the mean first birth interval. The effect size for parametric model is the time ratio. For each covariate, a time ratio value greater than one can be interpreted as individual experiences the event at a later timing. Conversely, a time ratio value of less than one implies that individuals will experience the event of interest faster. The statistical software used for modeling is Stata/SE, version 10 and R software used for plotting of graph.

## Results

**Figure 1:**
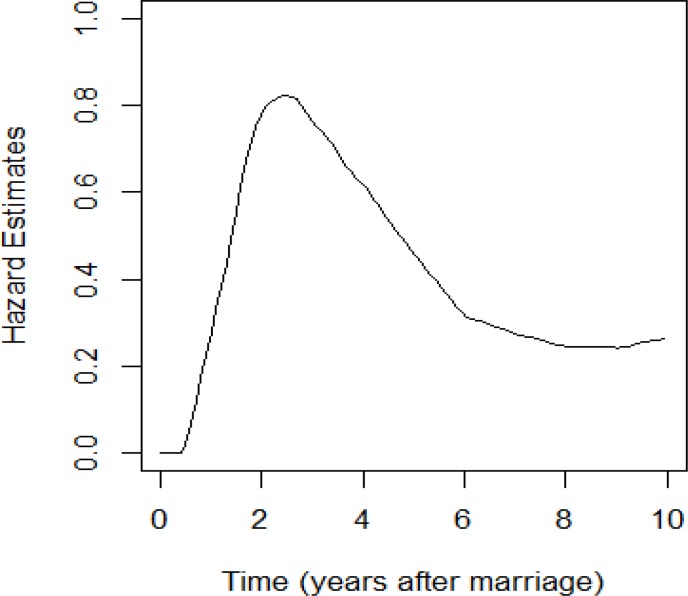
The live birth hazard in women with first pregnancy

Of 654 women, 621 (95%) had experienced first live birth, and the others (5%) were censored (right censored). The mean and median of the first live birth interval for women experiencing the event were 2.6 and 2.1 years, respectively. The mean age at marriage was 18.3±3.8 years. Proportion of women not having live birth before 1, 2, 3, 4, 5 years after marriage is 0.55, 0.23, 0.11, 0.07 and 0.05 percent. Table I shows the demographic characteristics of 654 women included in the study. We applied only the age at marriage in the analysis, because the age at marriage was highly correlated with the age at first pregnancy (r=0.9, p<0.001). The shape of hazard function is plotted in Figure 1. Up to two years after marriage, the hazard of live birth increases. After that, the hazard of live birth decreases. This shape, suggests the appropriateness of the Generalized Gamma and log-logistic models. 

The Generalized Gamma and log-logistic are more preferable when hazard rises to a peak before decreasing. By using the Log rank test, age at marriage of women had significant effects on the duration of birth interval after marriage (p=0.002). The median length of live birth interval after marriage decreases with the increase age at marriage. Menstrual status had significant effects on the duration of birth interval after marriage (p<0.001). In women that had irregular menstrual cycle, the median length of live birth interval after marriage was longer. 

We used the parametric models and Cox model after adjustment of other covariates. We find age at marriage of women, menstrual status and education level of women affect live birth interval after marriage. The results of the survival regression analysis are summarized in Table II. The significant prognostic factors identified by the Generalized Gamma, Log-logistic and the Cox model were identical. Based on the Generalized Gamma model, a one year increase in the marriage age decreases the time of the first live birth after marriage by 1%. In other words, the women have faster transition to first live birth (TR=0.99, p=0.006). By Cox model, when a one year increase in age at marriage, the hazard rate of live birth increases by 3% (HR=1.03, p=0.006). 

Based on the Generalized Gamma model, women with primary and secondary education had faster transition to first live birth compared to those without any formal education (TR=0.90, p=0.007) and by cox model, the hazard rate of live birth of women with primary and secondary education is 33% more than those without any formal education (HR=1.33, p=0.03). Based on the Generalized Gamma model, women with regular menses had faster transition to first live birth compared to those with irregular menses (TR=0.88, p=0.004) and by the Cox model, the hazard rate of live birth of women with regular menses is 68% more than those with irregular menses (HR=1.68, p<0.001). Moreover, the results are showed that the standard errors with parametric models are lower than the Cox model.

**Table I:** Demographic characteristics of women aged 15-49 years in rural areas of Shiraz

**Independent variables**		**No (%)**	**First birth interval (years)**		**Log Rank Test** **p-value** a
			**Mean ± SD**	**Median**	
Age at marriage (years)					
	≤ 15	122 (18.7%)	3.0 ± 2.0	2.44	0.002
	15-20	350 (53.6%)	2.5 ± 1.7	2.04	
	>20	182 (27.8%)	2.5 ± 1.8	1.96	
Age at menarche (years)					
	≤13	362 (56.2%)	2.6 ± 1.8	2.10	0.37
	>13	282 (43.8%)	2.6 ± 1.9	1.96	
Wives level of education					
	Illiterate	104 (15.9%)	2.8 ± 2.0	2.17	0.332
	Primary and middle school	474 (72.5%)	2.6 ± 1.9	2.04	
	High school and higher education	76 (11.6%)	2.5 ± 1.3	2.06	
Husbands level of education					
	Illiterate	86 (13.1%)	2.6 ± 1.5	2.04	0.983
	Primary and middle school	459 (79.2%)	2.6 ± 1.8	2.08	
	High school and higher education	109 (16.7%)	2.6 ± 2.1	2.06	
Income					
	Sufficient	290 (44.3%)	2.6 ± 2.0	2.06	0.714
	Insufficient	364 (55.7%)	2.6 ± 1.7	2.06	
Menstrual status					
	Regular	592 (90.5%)	2.5 ± 1.5	2.04	p<0.001
	Irregular	62 (9.5%)	3.7 ± 3.5	2.40	

a Survival analysis; Log Rank Test

**Table II:** The results of data analysis using survival analysis

**Models**		**Cox**		**Log-logistic**		**GG**	
**HR** a **(SE**^b^**)**	**p-value** c	**TR** d ** (SE)**	**p-value** **e**	**TR (SE)**	**p-value** e
Age at marriage		1.03 (0.01)	0.006	0.98 (0.01)	p<0.001	0.99 (0.01)	0.006
Income							
	Insufficient						
	Sufficient	0.94 (0.08)	0.46	1.02 (0.03)	0.58	1.02 (0.03)	0.40
Wives level of education							
	Illiterate						
	Primary and middle school	1.33 (0.17)	0.03	0.90 (0.04)	0.03	0.90 (0.03)	0.007
	High school and higher	1.23 (0.22)	0.24	0.97 (0.07)	0.63	0.95 (0.05)	0.40
Husband level of education							
	Illiterate						
	Primary and middle school	0.89 (0.12)	0.39	1.03 (0.05)	0.54	1.04 (0.04)	0.35
	High school and higher	0.89 (0.15)	0.49	1.03 (0.07)	0.64	1.06 (0.07)	0.26
Age at menarche		1.01 (0.03)	0.67	1.00 (0.01)	0.91	1.01 (0.01)	0.72
Menstrual status							
	Irregular						
	Regular	1.68 (0.24)	p<0.001	0.82 (0.05)	0.001	0.88 (0.04)	0.004
Log-likelihood				-342.1		-290.7	
AIC				704.3		603.5	

a HR: Hazard Ratio

b SE: Standard Error

c Cox regression

d TR: Time Ratio

e Parametric survival analysis

## Discussion

The timing of the first birth is known to be a critical factor in completed family size. First childbirth has a direct relationship with number of factors such as couples’ educational and occupational status, age at marriage, etc. (19). Few studies have been carried out on the timing between age at marriage and the first birth, especially in Iran. Our purpose was to identify prognostic factors of transition to first birth using parametric models and semi-parametric method (Cox model). The major advantage of using survival models in analyzing birth interval data, especially first birth, is using all of data, even women that are censored. But, in other statistical methods these data must be excluded. In our data, the percentage of censoring was 5%. A good discrimination among parametric models requires the censoring percentage not to exceed 40-50% (20). 

As our results indicated, age at marriage, women's education and menstrual status were significant prognostic factors of transition to the first live birth. The result showed that women who married at a relatively later time had a significantly shorter timing to first birth. Age at first marriage is an important variable in most fertility studies since it indicates the beginning of a woman’s exposure to sexual intercourse and hence to the risk of childbearing (1, 21-23). Slow rate of population growth is related to older age at first marriage or longer birth interval between marriage and first birth (19, 22). Age at first marriage and first birth determine the health of a mother and a child. 

In the present study, women with primary and secondary education have faster transition to first live birth. Women's education is considered to be an essential component of reproductive behavior. When women spend a longer time at school, this is likely to significantly affect both age at marriage and the duration between marriage and the first birth. Our results agree with the findings of other studies (1, 9-10). In addition, menstrual status was one of the significant variables in this study. Women with regular menstrual cycle had faster transition to first live birth.

According to the results of this study, the parametric survival model is more suitable to analysis this type data to several reason. 1) The hazard function shape showed that using parametric models are appropriate. 2) Table II showed that standard error of coefficients with parametric models is lower than cox model. 3) Time ratio is more appropriate to evaluate the direct effect of variables on the first birth interval compare to hazard ratio. In general, because the first childbirth is an important event in the reproductive life of a woman, further research is needed to be carried out to explore the influential factors affecting the length of the first birth interval. Moreover, for the sake of increasing precision, relevant analyses such as parametric survival analysis must be proposed.
